# Dietary fatty acids and risk of non-alcoholic steatohepatitis: A national study in the United States

**DOI:** 10.3389/fnut.2022.952451

**Published:** 2022-07-26

**Authors:** Xiao-Ting Lu, Yong-Dong Wang, Ting-Ting Zhu, Hui-Lian Zhu, Zhao-Yan Liu

**Affiliations:** ^1^Department of Nutrition, School of Public Health, Sun Yat-sen University, Guangzhou, China; ^2^Guangdong Provincial Key Laboratory of Food, Nutrition and Health, School of Public Health, Sun Yat-sen University, Guangzhou, China; ^3^Department of Internal Medicine, Shaoguan First People’s Hospital, Shaoguan, China; ^4^Department of Food Science and Engineering, School of Food Science and Engineering, Hainan Tropical Ocean University, Sanya, China

**Keywords:** polyunsaturated fatty acids, non-alcoholic steatohepatitis, subtypes of fatty acids, dietary fatty acids, national study, national health and nutrition examination survey

## Abstract

**Background:**

Non-alcoholic steatohepatitis (NASH), the early invertible stage of non-alcoholic fatty liver disease, has become a public health challenge due to the great burden and lack of effective treatment. Dietary nutrients are one of the modifiable factors to prevent and slow down disease progression. However, evidence linking dietary fatty acids intake and risk of NASH is lacking.

**Objectives:**

This study aimed to examine the association between dietary total saturated fatty acids (SFAs), monounsaturated fatty acids (MUFAs), polyunsaturated fatty acids (PUFAs), their subtypes, the ratio of unsaturated (UFAs) to SFAs, and the risk of NASH among a nationwide population in the United States.

**Methods:**

This cross-sectional study was conducted among 4,161 adults in the national health and nutrition examination survey in 2017–2018 cycle. Moreover, NASH was defined by transient elastography. Dietary fatty acids were assessed using a validated 24-h food recall method. Logistic regression models were used to estimate odds ratios (ORs) and 95% confidence intervals (95% CIs).

**Results:**

A total of 2,089 (50.2%) participants with NASH were identified. Compared with participants in the bottom tercile of dietary intakes of total PUFAs, those in the highest tercile had lower risk of NASH, with an adjusted OR of 0.67 (95% CI: 0.46–0.97). Similar associations were found between the subtype of PUFA 18:3 and NASH, while the fully adjusted OR in the highest tercile was 0.67 (95% CI: 0.47–0.96). Interactions of dietary PUFAs and body mass index (BMI) could be found influencing NASH risk. Stronger associations of dietary total PUFAs intakes with NASH risk were found in obese participants (OR, 95% CI: 0.41, 0.22–0.75) than in the non-obese participants (OR, 95% CI: 1.00, 0.70–1.43; *p*-interaction = 0.006). Similar effects on risk of NASH were also observed between BMI and dietary intakes of PUFA 18:3. However, no significant associations were observed between NASH risk and dietary total SFAs, MUFAs, their subtypes as well as the ratio of UFAs to SFAs.

**Conclusion:**

Dietary intakes of total PUFAs, as well as its subtype of PUFA 18:3, were inversely associated with risk of NASH. The further large prospective studies need to be conducted to confirm the findings of this study.

## Introduction

Non-alcoholic fatty liver disease (NAFLD) has emerged as one of the most common causes of chronic liver disease, as well as the most quickly growing promoters to liver morbidity and mortality in the United States (1). The prevalence of NAFLD has increased rapidly over the past three decades (1, 2) and the number of people suffering from NAFLD are expected to be more than 100 million by 2030 (3). Usually, parallels to the prevalence of obesity, type 2 diabetes and cardiovascular disease, NAFLD decreases life expectancy and increases requirements of liver transplantation (4, 5). Moreover, NAFLD progresses from steatosis, non-alcoholic steatohepatitis (NASH), to liver fibrosis and even ultimately to hepatocellular carcinoma (4), while approximately 20–30% of individuals may progress to NASH (6). It is estimated that NASH prevalence will increase up to 56% by 2030 in the United States at a greater rate than the other European and Asian countries (3). Due to the persistent cellular damage and excess fat deposition, patients with NASH are more likely to progress to the irreversible advanced fibrosis (4). Given the great burden as well as the lack of effective treatment, it is important to prevent and slow down disease progression at the early stage. The dietary nutrients are considered to be one of the effective and modifiable factors (7).

Fatty acids are composed of saturated fatty acids (SFAs) and unsaturated fatty acids (UFAs), the latter includes monounsaturated fatty acids (MUFAs) and polyunsaturated fatty acids (PUFAs). They can also be subdivided into a number of subtypes according to the position and numbers of carbon atoms and double bonds. Fatty acids play an important and indispensable role in human diet and body metabolism, which not only are sources of essential fatty acids and precursors of bioactive substances but also involve in bio-membrane structure formation, signal transmission, and lipid transportation. Liver is the main metabolic organ of fatty acids. Improper dietary fatty acids consumption or *de novo* lipogenesis beyond liver capacity has the consequences of abnormal cellular lipid composition and toxic lipid accumulation, leading to organelle dysfunction, cellular damage, inflammation, and occurrence of diseases (8). However, the results from epidemiological studies linking the relationships between dietary fatty acids and NAFLD are sparse and contradictory. For example, a systematic review reported that patients with NAFLD had disturbed fatty acids metabolisms compared to healthy controls (6). Evidences from the previous studies demonstrated that people with NASH possessed elevated total SFAs, while total MUFAs and PUFAs may be protective (9). However, a non-linear association between total PUFAs intakes and NAFLD was found among Chinese Han adults, the total PUFAs was positively associated with risk of NAFLD at certain dose of total PUFAs consumption (10). The reasons for the discrepancies could be ascribed to differences of sample sizes, population, and different detect methods of NAFLD.

Liver biopsy, the gold standard of NAFLD assessments, is unsuitable for population screening due to its invasion. Other methods of detection such as hepatic steatosis index (11), fatty liver index (12), NAFLD liver fat score (13), SteatoTest (14), which are calculated by individual conditions and blood biomarkers, have several shortages such as limited sensitivity, specificity, popularization or expensive price. A non-invasive and effective approach to distinguish NAFLD as well as its stages is urgently needed. Compared with liver biopsy, transient elastography is a non-invasive, convenient and fast method to assess NAFLD from NASH to liver fibrosis, but few studies apply it to diagnosis of NAFLD in a large population.

Furthermore, different fatty acids subtypes have different effects on liver health. For example, as for hepatic fatty acids compositions, a decrease in the ratio of SFA 18:0 to SFA 16:0 was associated with the steatosis score and insulin resistance, while higher ratio of MUFA 16:1 to SFA 16:0 was associated with lobular inflammation and hepatocellular ballooning in patients with NASH (15). However, none of the existing studies have investigated the association between different subtypes of dietary fatty acids and the risk of NAFLD. Therefore, in this study, we aimed to examine the association between dietary total SFAs, MUFAs, PUFAs, their subtypes, the ratio of UFAs to SFAs, and the risk of NASH (the early invertible stage of NAFLD), which was determined by liver ultrasound transient elastography among a nationwide population in the United States.

## Materials and methods

### Study population

**FIGURE 1:**
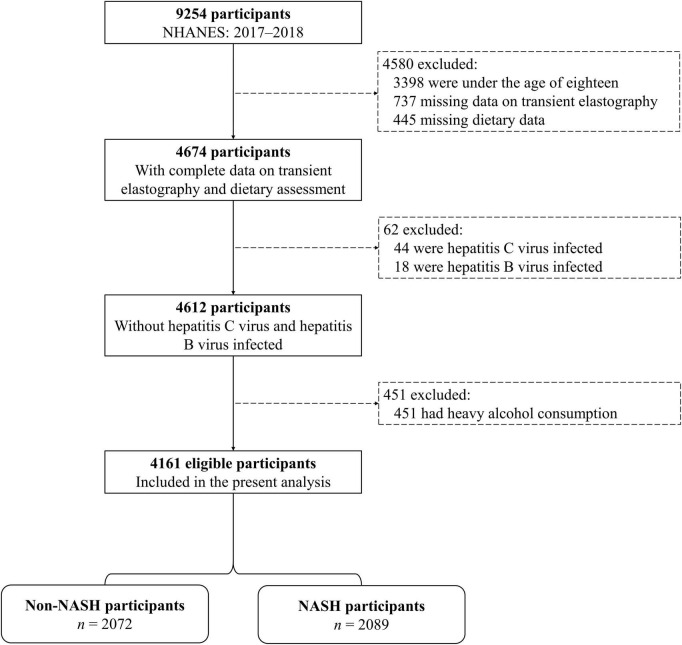
Flowchart of selection of participants from national health and nutrition examination survey (NHANES). Abbreviations: NASH, non-alcoholic steatohepatitis.

The analysis of this study was conducted based on data from national health and nutrition examination survey (NHANES), which was carried out by the Center for Disease Control and Prevention of the United States. Detailed information could be found from the official website^1^. In this study, we included participants in the 2017–2018 NHANES cycle (*n* = 9,254). Participants were excluded if they were underage (<18 years, *n* = 3,398), had unavailable data on transient elastography (*n* = 737) or dietary assessment (*n* = 445), were hepatitis C virus (*n* = 44) or hepatitis B virus (*n* = 18) infected, or had heavy alcohol consumption (>30 g/day for men and >20 g/day for women, *n* = 451). Finally, 4,161 participants were included (Figure 1). Due to the discrepancies between adults and minors in many aspects, we only compare the characteristics of adults (≥18 years) in the included and the excluded participants. The included participants did not differ by most of the basic characteristics from adults excluded from this analysis (*n* = 1,695, Supplementary Table 1). The institutional review board approval of the National Center for Health Statistics and informed consent was obtained from all participants before data collection.

### Assessment and definition of non-alcoholic steatohepatitis

The liver ultrasound transient elastography was first used to provide objective measures for hepatic steatosis in the NHANES Mobile Examination Center (MEC) in the 2017–2018 cycle (16). The participants who were aged ≥12 years, were able to lie down, were not pregnant, had no implanted electronic medical device, and had no lesions where measurements would be taken were eligible to test. Using the FibroScan model 502 V2 Touch equipped with a medium or extra-large probe, controlled attenuation parameter (CAP) was measured and recorded as the indicator for hepatic steatosis according to the fatness in the liver. The elastography exam was performed by well-trained NHANES health technicians according to the manufacture guidelines. The inter-rater reliability between health technician and reference examiners was 0.94 (mean differences 4.5 ± 19.8 dB/m), and variances within and between the device machines and the probes over time were under control (intra-machine coefficient of variation was 1.2–3.2%; inter-machine intra class correlation was 2–22%) for CAP. High accuracy of the CAP measurements for the detection of steatosis compared to biopsy has been reported in the previous studies (17–19).

In this study, those CAP scores equal or greater than 263 dB/m (had a 96% positive predictive value) were defined as cases of NASH (20), others as controls (non-NASH). The cut-off point was determined before statistical analysis.

### Assessment of dietary fatty acids

Assessment of dietary intakes was undertaken by trained interviewers using a validated 24-h food recall method in the MEC, and was repeated by telephone 3–10 days later. Values of dietary intakes in these two discrete days were averaged to represent their dietary status, while values of the first time were used for participants with a lack of dietary data of the second time (12.2% in the current analysis). The intakes of total energy, SFAs, MUFAs, PUFAs, and their subtypes were estimated according to the United States Department of Agriculture’s Food and Nutrient Database for Dietary Studies. The healthy eating index-2015 (HEI-2015) was calculated according to the MyPyramid Equivalents Database 2.0 for United States Department of Agriculture Survey Foods to reflect the quality of diet comprehensively (Supplementary Table 2). A higher score between 0–100 indicated a better dietary quality. The ratio of UFAs to SFAs was calculated as (PUFAs + MUFAs)/SFAs, and therefore divided into three levels according to the scoring standards of HEI-2015.

### Covariates collection

Sociodemographic information including age, sex (men and women), ethnicity (non-Hispanic white, non-Hispanic black, Mexican American, and others), marital status (married/living with partner, separated/divorced/widowed, and never married), education levels (less than high school, high school or equivalent, and college or above), and family income-to-poverty ratio (<1.3, 1.3–3.5, and >3.5) were collected. Anthropometric measurements including height, weight, and waist circumference were measured. Body mass index (BMI) was calculated as weight (kg)/height squared (m^2^). Never smokers were participants who smoked less than 100 cigarettes in their lifetime. Former smokers were those who had given up smoking before the interview, and current smokers were those who smoked more than 100 cigarettes in their whole life and kept the habit of smoking at the time of interview. The participants who had regular exercise were defined as those who reported that they had moderate- or vigorous-intensity physical activities at least 10 min in a typical week, in succession with small or large increases in heart rate or breathing. Those who were exposed to oral corticosteroid medication for more than 180 days were defined as the presence of use of oral corticosteroid. Hypertension was defined as elevated blood pressure (systolic/diastolic blood pressure equal or higher than 140/90 mm Hg), self-reported hypertension diagnosis by clinician or taking anti-hypertensive drugs. Diabetes mellitus was defined as fasting plasma glucose concentration ≥ 7.0 mmol/L, glycosylated hemoglobin level ≥ 6.5%, self-reported diabetes diagnosis, or use of diabetic pills (including insulin). Dyslipidemia was defined if any of the following status was matched: (1) Total cholesterol ≥ 200 mg/dl, (2) triglyceride ≥ 150 mg/dl, (3) low-density lipoprotein cholesterol ≥ 130 mg/dl, (4) high-density lipoprotein cholesterol < 40 mg/dl or <50 mg/dl for men and women, respectively, (5) self-reported taking prescribed lipid-modifying medication. Cardiovascular disease was defined as self-reported diagnosis of angina, congestive heart failure, coronary heart disease, heart attack, or stroke. Cancer was defined as self-reported diagnosis of any kind of cancer by clinician during the whole lifetime.

Three consecutive blood pressure measurements were obtained after resting quietly in a seated position for 5 min in the MEC according to the physician examination procedures manual (21). A fourth determination would be taken if a blood pressure measurement was interrupted or incomplete. The values of these three or four readings were averaged to represent their blood pressure status. Blood collection took place in the MEC under standardized conditions at each survey location including collecting, processing, storing, and shipping. Laboratory parameters including alanine aminotransferase (ALT), alkaline phosphatase (ALP), aspartate aminotransferase (AST), and gamma-glutamyl transferase (GGT), glucose, glycosylated hemoglobin, total cholesterol, triglyceride, low-density lipoprotein cholesterol, and high-density lipoprotein cholesterol were measured using corresponding methods described in the official website (22).

### Statistical analysis

Given to the complex sampling design, appropriate sample weight was conducted according to NHANES analytic guidelines in the current analysis. Non-normally distributed data were natural logarithm transformed and estimates of fatty acids intakes were adjusted for energy intakes using the residuals method (23) before further analysis. The basic characteristics and dietary intakes of fatty acids of participants (overall, non-NASH, and NASH) were described by the weighted mean and standard error (SE) for continuous variables, as well as counts and weighted frequencies for categorical variables. The differences of basic characteristics between participants with and without NASH were compared by general linear models and Chi-squared test as appropriate. Analyses of covariance (ANCOVA) controlling for sex, age, and BMI were used for comparison of the mean differences in dietary intakes of energy and fatty acids.

Participants were divided into three groups according to terciles in the non-NASH group of dietary intakes of total SFAs, MUFAs, PUFAs and their subtypes. Logistic regression models were performed to examine the association between the dietary intakes of fatty acids and the risk of NASH. According to the previous studies (24) and the specialized knowledge, several covariates were selected for adjustments to minimize the residual confounding. Minimally adjusted models included age (continuous), sex (categorized), and BMI (continuous). Other potential risk factors, including ethnicity (categorized), marital status (categorized), education levels (categorized), family income-to-poverty ratio (categorized), waist circumference (continuous), smoking status (categorized), regular exercise (categorized), use of oral corticosteroid (categorized), HEI-2015 (continuous), ALT (continuous), ALP (continuous), AST (continuous), GGT (continuous), prevalence of hypertension, diabetes mellitus, dyslipidemia, cardiovascular disease, and cancer (categorized) were additionally adjusted in the fully adjusted models. Odds ratios (ORs) and 95% confidence intervals (CIs) were calculated with the lowest terciles as the reference. Since only a few covariates were missing with a small portion, observations with missing data were automatically excluded from the corresponding adjusted models. Sensitivity analysis was conducted among participants without use of oral corticosteroid (*n* = 4,118). Stratified analysis was performed to examine whether the association between terciles of dietary fatty acids intakes and risk of NASH was different in participants with various characteristics. Interactions were estimated by including the multiplicative interaction terms.

The data were analyzed from January 2022 to May 2022. Statistical analyses were performed using the R software 4.1.0 (the “survey” package) and the SPSS, version 25.0 (IBM Corp., Armonk, NY, United States). All tests were two-sided and *p* < 0.05 was considered statistically significant.

## Results

### Basic characteristics and dietary intakes of fatty acids of study participants

Weighted distributions of sociodemographic information, lifestyle, laboratory parameters, and prevalence of several chronic diseases for overall population, participants with and without NASH were shown in Table 1. Of the 4,161 study participants, 48.6% were men and mean (SE) age was 47.5 (0.8) years. The participants in the current study tended to be obese with a mean BMI of 29.8 (0.3) kg/m^2^, and a total of 2,089 (50.2%) participants with NASH were identified. Compared with non-NASH participants, those with NASH were more likely to be older, had a higher BMI, higher waist circumference, higher levels of ALT, ALP, AST, and GGT, higher prevalence of hypertension, diabetes mellitus, dyslipidemia, cardiovascular disease, and cancer, had lower education levels, and lower frequencies of regular exercise (*p* < 0.05). A significantly greater proportion of participants with NASH were men, were Mexican American, were married, and were former or current smokers (*p* < 0.05). No significant differences were observed in family income-to-poverty ratio and use of oral corticosteroid between participants with and without NASH (*p* > 0.05).

**TABLE 1:** Basic characteristics of participants of this study.

	Overall (*n* = 4,161)	Non-NASH (*n* = 2,072)	NASH (*n* = 2,089)	*p*
Age, years	47.5 ± 0.8	43.8 ± 0.9	51.3 ± 0.8	<0.001
Sex, *n* (%)				<0.001
Men	2,015 (48.6)	915 (43.9)	1,100 (53.7)	
Women	2,146 (51.4)	1,157 (56.1)	989 (46.3)	
Ethnicity, *n* (%)				<0.001
Non-Hispanic white	1,442 (62.6)	695 (63.0)	747 (62.1)	
Non-Hispanic black	971 (11.4)	571 (13.3)	400 (9.3)	
Mexican American	582 (9.1)	205 (6.3)	377 (12.0)	
Others	1,166 (17.0)	601 (17.4)	565 (16.6)	
Marital status, *n* (%)				<0.001
Married/living with partner	2,340 (60.0)	1,056 (53.1)	1,284 (67.4)	
Separated/divorced/widowed	885 (18.0)	424 (18.1)	461 (17.9)	
Never married	711 (18.1)	426 (22.9)	285 (12.9)	
Education levels, *n* (%)				0.002
Less than high school	753 (10.4)	350 (9.5)	403 (11.3)	
High school or equivalent	956 (27.0)	456 (25.1)	500 (29.0)	
College or above	2,225 (58.7)	1,099 (59.4)	1,126 (58.0)	
Family income-to-poverty ratio, *n* (%)				0.092
<1.3	1,039 (20.0)	543 (20.7)	496 (19.1)	
1.3–3.5	1,552 (37.6)	732 (34.7)	820 (40.7)	
>3.5	1,068 (42.4)	547 (44.6)	521 (40.1)	
BMI, kg/m^2^	29.8 ± 0.3	26.3 ± 0.3	33.6 ± 0.3	<0.001
Waist circumference, cm	100.7 ± 0.8	91.3 ± 0.9	110.9 ± 0.8	<0.001
Smoking status, *n* (%)				0.001
Never smoker	2,517 (60.2)	1,303 (63.1)	1,214 (57.1)	
Former smoker	958 (24.1)	386 (19.7)	572 (28.7)	
Current smoker	686 (15.7)	383 (17.2)	303 (14.1)	
Regular exercise, *n* (%)	1,980 (53.8)	1,097 (60.7)	883 (46.5)	<0.001
Use of oral corticosteroid ≥ 180 days, *n* (%)	43 (0.9)	22 (0.9)	21 (0.9)	0.949
Laboratory parameters, IU/L				
ALT	22.5 ± 0.3	19.1 ± 0.3	26.1 ± 0.6	<0.001
ALP	77.4 ± 0.7	74.2 ± 1.2	80.8 ± 0.9	0.001
AST	21.5 ± 0.2	20.8 ± 0.3	22.4 ± 0.4	0.014
GGT	27.8 ± 0.6	22.5 ± 0.7	33.4 ± 0.9	<0.001
Prevalence of chronic diseases, *n* (%)				
Hypertension	1,854 (38.3)	722 (26.2)	1,132 (51.2)	<0.001
Diabetes mellitus	877 (15.4)	218 (5.6)	659 (25.9)	<0.001
Dyslipidemia	2,753 (65.0)	1,121 (53.2)	1,632 (77.7)	<0.001
Cardiovascular disease	442 (8.3)	166 (5.3)	276 (11.6)	<0.001
Cancer	415 (10.6)	177 (9.1)	238 (12.1)	<0.001

NASH, non-alcoholic steatohepatitis; BMI, body mass index; ALT, alanine aminotransferase; ALP, alkaline phosphatase; AST, aspartate aminotransferase; and GGT, gamma-glutamyl transferase.

Data were presented as weighted mean ± SE or counts (weighted frequencies).

With regard to dietary intakes of overall participants, energy intakes controlling for sex, age, and BMI were 1,980.53 (12.21) kcal/day. Mean (SE) of dietary total SFAs, MUFAs and PUFAs adjusted for energy intakes, sex, age, and BMI were 23.21 (0.11) g/day, 24.97 (0.11) g/day, and 17.21 (0.09) g/day (Table 2). Compared with non-NASH participants, those with NASH had lower dietary intakes of total MUFAs, total PUFAs, MUFA 18:1, PUFA 18:2, PUFA 20:4, and lower score in HEI-2015 (*p* < 0.05). Other dietary intakes including energy intake, total SFAs, the ratio of UFAs and SFAs, and other 16 subtypes of fatty acids did not show significant difference between participants with and without NASH.

**TABLE 2:** Dietary fatty acids intakes of participants of this study.

Dietary intakes	Overall (*n* = 4,161)	Non-NASH (*n* = 2,072)	NASH (*n* = 2,089)	*p*
Energy intake, kcal/day^a^	1,980.53 ± 12.21	1,974.64 ± 18.68	1,986.43 ± 18.52	0.674
Total SFAs, g/day^b^	23.21 ± 0.11	23.20 ± 0.16	23.23 ± 0.16	0.909
Total MUFAs, g/day^b^	24.97 ± 0.11	25.23 ± 0.16	24.72 ± 0.16	0.036
Total PUFAs, g/day^b^	17.21 ± 0.09	17.48 ± 0.14	16.95 ± 0.14	0.015
The ratio of UFAs to SFAs^c^				0.936
≤1.2	453 (12.0)	226 (11.8)	227 (12.2)	
1.2–2.5	3,070 (76.1)	1,508 (76.1)	1,562 (76.0)	
≥2.5	637 (12.0)	337 (12.1)	300 (11.8)	
Subtypes of fatty acids, g/day^b^				
SFA 4:0	0.394 ± 0.004	0.390 ± 0.007	0.397 ± 0.007	0.492
SFA 6:0	0.261 ± 0.003	0.261 ± 0.004	0.262 ± 0.004	0.819
SFA 8:0	0.223 ± 0.003	0.224 ± 0.004	0.222 ± 0.004	0.705
SFA 10:0	0.441 ± 0.004	0.441 ± 0.007	0.441 ± 0.007	0.988
SFA 12:0	0.805 ± 0.016	0.818 ± 0.025	0.793 ± 0.024	0.498
SFA 14:0	1.890 ± 0.016	1.880 ± 0.024	1.900 ± 0.024	0.591
SFA 16:0	12.875 ± 0.054	12.866 ± 0.083	12.884 ± 0.082	0.886
SFA 18:0	5.489 ± 0.028	5.480 ± 0.043	5.499 ± 0.042	0.763
MUFA 16:1	1.024 ± 0.008	1.035 ± 0.012	1.013 ± 0.012	0.207
MUFA 18:1	23.421 ± 0.099	23.658 ± 0.152	23.185 ± 0.151	0.038
MUFA 20:1	0.286 ± 0.003	0.290 ± 0.005	0.281 ± 0.005	0.198
MUFA 22:1	0.033 ± 0.002	0.034 ± 0.003	0.032 ± 0.003	0.536
PUFA 18:2	15.231 ± 0.085	15.469 ± 0.130	14.993 ± 0.129	0.015
PUFA 18:3	1.621 ± 0.012	1.641 ± 0.018	1.601 ± 0.018	0.140
PUFA 18:4	0.013 ± 0.001	0.012 ± 0.001	0.013 ± 0.001	0.653
PUFA 20:4	0.148 ± 0.002	0.152 ± 0.002	0.144 ± 0.002	0.034
PUFA 20:5	0.033 ± 0.001	0.032 ± 0.002	0.033 ± 0.002	0.744
PUFA 22:5	0.025 ± 0.001	0.026 ± 0.001	0.024 ± 0.001	0.368
PUFA 22:6	0.072 ± 0.003	0.071 ± 0.004	0.073 ± 0.004	0.764
HEI-2015	51.2 ± 0.8	52.2 ± 0.9	50.1 ± 0.7	0.008

NASH, non-alcoholic steatohepatitis; SFAs, saturated fatty acids; MUFAs, monounsaturated fatty acids; PUFAs, polyunsaturated fatty acids; UFAs, unsaturated fatty acids; and HEI-2015, healthy eating index-2015.

Data were presented as mean ± SE or counts (weighted frequencies).

^a^Data were assessed with ANCOVA controlling for sex, age, and BMI.

^b^Dietary intakes of fatty acids were adjusted for energy intakes using the residuals method first, and then were assessed with ANCOVA controlling for sex, age, and BMI.

^c^The ratio of UFAs to SFAs was calculated as (PUFAs + MUFAs)/SFAs.

### Dietary intakes of fatty acids and risk of non-alcoholic steatohepatitis

Associations between terciles of dietary total SFAs, MUFAs, PUFAs, their subtypes, the ratio of UFAs to SFAs and NASH were presented in Table 3. An inverse association between dietary total PUFAs and NASH risk was found, with an OR of 0.67 (95% CI: 0.46–0.97) at the highest tercile in comparison with the bottom tercile after adjustments for potential risk factors. Similar associations were found between the subtype of PUFAs 18:3 and NASH, while the fully adjusted OR in the highest tercile was 0.67 (95% CI: 0.47–0.96). Participants who were in the second tercile of total MUFAs and MUFA 18:1, rather than in the highest tercile, had a lower risk of NASH compared with the first tercile after full adjustments. However, dietary intakes of total SFAs, other subtypes, as well as the ratio of UFAs to SFAs did not show significant associations with NASH risk after adjustment for potential risk factors in any tercile. Moreover, results remained largely unchanged in the sensitivity analysis when restricting participants to those who did not use oral corticosteroid (Supplementary Table 3).

**TABLE 3:** Odds ratios (ORs) and 95% confidence intervals (CIs) of non-alcoholic steatohepatitis by terciles of dietary intakes of fatty acids among controls (*n* = 4,161).

Dietary intakes	NASH/non-NASH	Levels, g/day^b^	Model 1	Model 2	Model 3
Total SFAs					
T1	632/690	≤19.78	1.00	1.00	1.00
T2	745/691	19.78–25.56	1.08 (0.84, 1.39)	1.04 (0.78, 1.39)	0.97 (0.69, 1.38)
T3	712/690	>25.56	1.10 (0.86, 1.40)	0.86 (0.61, 1.21)	0.79 (0.53, 1.17)
Total MUFAs					
T1	717/690	≤22.09	1.00	1.00	1.00
T2	669/691	22.09–27.23	0.79 (0.60, 1.04)	0.69 (0.51, 0.94)	0.69 (0.49, 0.96)
T3	703/690	>27.23	0.90 (0.71, 1.15)	0.63 (0.44, 0.91)	0.67 (0.44, 1.01)
Total PUFAs					
T1	722/690	≤14.34	1.00	1.00	1.00
T2	708/691	14.34–19.24	0.92 (0.68, 1.24)	0.74 (0.51, 1.09)	0.77 (0.48, 1.23)
T3	659/690	>19.24	0.76 (0.57, 1.02)	0.62 (0.47, 0.81)	0.68 (0.48, 0.96)
The ratio of UFAs to SFAs^a^					
≤1.2	227/226	–	1.00	1.00	1.00
1.2–2.5	1,562/1,508	–	0.97 (0.74, 1.26)	0.91 (0.68, 1.22)	1.05 (0.68, 1.60)
≥2.5	300/337	–	0.94 (0.65, 1.38)	0.89 (0.70, 1.12)	1.08 (0.69, 1.68)
Subtypes of fatty acids					
SFA 4:0					
T1	641/685	≤0.234	1.00	1.00	1.00
T2	774/687	0.234–0.462	1.17 (0.92, 1.49)	1.30 (0.98, 1.71)	1.28 (0.97, 1.69)
T3	664/685	>0.462	0.98 (0.73, 1.32)	1.08 (0.75, 1.54)	1.11 (0.73, 1.70)
SFA 6:0					
T1	665/682	≤0.161	1.00	1.00	1.00
T2	744/682	0.161–0.307	1.07 (0.87, 1.30)	1.13 (0.86, 1.48)	1.07 (0.81, 1.41)
T3	666/682	>0.307	0.96 (0.69, 1.34)	0.97 (0.67, 1.40)	1.01 (0.67, 1.52)
SFA 8:0					
T1	688/688	≤0.140	1.00	1.00	1.00
T2	706/689	0.140–0.247	0.92 (0.74, 1.13)	0.98 (0.75, 1.28)	1.03 (0.76, 1.40)
T3	690/688	>0.247	0.99 (0.73, 1.32)	1.05 (0.77, 1.43)	1.15 (0.81, 1.62)
SFA 10:0					
T1	668/690	≤0.290	1.00	1.00	1.00
T2	762/689	0.290–0.518	1.06 (0.89, 1.25)	1.13 (0.89, 1.44)	1.14 (0.85, 1.52)
T3	658/690	>0.518	0.91 (0.68, 1.20)	1.01 (0.73, 1.41)	1.07 (0.71, 1.60)
SFA 12:0					
T1	697/689	≤0.404	1.00	1.00	1.00
T2	689/690	0.404–0.783	0.95 (0.73, 1.23)	1.00 (0.73, 1.37)	0.93 (0.61, 1.41)
T3	703/689	>0.783	0.96 (0.70, 1.33)	1.02 (0.78, 1.34)	1.02 (0.78, 1.32)
SFA 14:0					
T1	655/690	≤1.344	1.00	1.00	1.00
T2	734/691	1.344–2.163	1.01 (0.78, 1.31)	1.03 (0.77, 1.36)	0.99 (0.76, 1.28)
T3	700/690	>2.163	1.00 (0.74, 1.35)	0.93 (0.68, 1.28)	0.90 (0.63, 1.28)
SFA 16:0					
T1	638/690	≤11.242	1.00	1.00	1.00
T2	724/691	11.242–14.124	1.11 (0.78, 1.56)	0.96 (0.66, 1.40)	0.98 (0.63, 1.51)
T3	727/690	>14.124	1.10 (0.85, 1.44)	0.78 (0.56, 1.09)	0.72 (0.50, 1.04)
SFA 18:0					
T1	621/690	≤4.593	1.00	1.00	1.00
T2	706/691	4.593–6.065	1.02 (0.76, 1.38)	0.91 (0.67, 1.23)	0.89 (0.61, 1.30)
T3	762/690	>6.065	1.20 (0.92, 1.56)	0.84 (0.59, 1.20)	0.74 (0.47, 1.15)
MUFA 16:1					
T1	667/690	≤0.775	1.00	1.00	1.00
T2	696/691	0.775–1.132	1.09 (0.87, 1.35)	0.90 (0.68, 1.18)	0.97 (0.68, 1.38)
T3	726/690	>1.132	1.23 (0.97, 1.56)	0.84 (0.60, 1.16)	0.84 (0.56, 1.27)
MUFA 18:1					
T1	717/690	≤20.691	1.00	1.00	1.00
T2	677/691	20.691–25.586	0.79 (0.60, 1.06)	0.67 (0.49, 0.92)	0.65 (0.46, 0.93)
T3	695/690	>25.586	0.95 (0.75, 1.19)	0.66 (0.47, 0.92)	0.70 (0.48, 1.01)
MUFA 20:1					
T1	705/690	≤0.205	1.00	1.00	1.00
T2	676/691	0.205–0.296	0.99 (0.76, 1.30)	0.82 (0.62, 1.08)	0.84 (0.63, 1.13)
T3	707/690	>0.296	1.02 (0.78, 1.34)	0.73 (0.52, 1.01)	0.77 (0.52, 1.15)
MUFA 22:1					
T1	659/651	≤0.008	1.00	1.00	1.00
T2	629/652	0.008–0.023	1.04 (0.87, 1.24)	0.92 (0.77, 1.10)	1.08 (0.84, 1.39)
T3	692/651	>0.023	1.13 (0.97, 1.32)	0.94 (0.68, 1.29)	1.08 (0.75, 1.56)
PUFA 18:2					
T1	728/690	≤12.745	1.00	1.00	1.00
T2	695/691	12.745–17.031	0.90 (0.65, 1.25)	0.73 (0.47, 1.12)	0.74 (0.45, 1.22)
T3	666/690	>17.031	0.78 (0.57, 1.05)	0.64 (0.47, 0.86)	0.69 (0.47, 1.00)
PUFA 18:3					
T1	690/690	≤1.244	1.00	1.00	1.00
T2	710/691	1.244–1.776	1.10 (0.80, 1.49)	1.01 (0.71, 1.43)	1.08 (0.73, 1.61)
T3	689/690	>1.776	0.85 (0.62, 1.15)	0.66 (0.49, 0.89)	0.68 (0.48, 0.95)
PUFA 18:4					
T1	500/479	≤0.001	1.00	1.00	1.00
T2	518/479	0.001–0.004	0.97 (0.66, 1.42)	0.91 (0.62, 1.35)	0.92 (0.58, 1.46)
T3	467/479	>0.004	1.11 (0.75, 1.65)	1.05 (0.66, 1.66)	1.01 (0.61, 1.69)
PUFA 20:4					
T1	657/688	≤0.093	1.00	1.00	1.00
T2	694/688	0.093–0.164	1.00 (0.71, 1.42)	0.83 (0.54, 1.26)	0.77 (0.48, 1.26)
T3	736/688	>0.164	1.20 (0.94, 1.54)	0.85 (0.63, 1.15)	0.75 (0.53, 1.08)
PUFA 20:5					
T1	654/664	≤0.006	1.00	1.00	1.00
T2	724/664	0.006–0.013	1.19 (0.95, 1.49)	1.05 (0.87, 1.27)	1.02 (0.82, 1.27)
T3	638/664	>0.013	1.11 (0.83, 1.49)	0.94 (0.65, 1.35)	0.99 (0.68, 1.43)
PUFA 22:5					
T1	678/673	≤0.014	1.00	1.00	1.00
T2	680/674	0.014–0.023	1.04 (0.73, 1.48)	0.93 (0.64, 1.36)	0.85 (0.59, 1.23)
T3	696/673	>0.023	1.09 (0.82, 1.45)	0.83 (0.58, 1.19)	0.83 (0.55, 1.23)
PUFA 22:6					
T1	603/644	≤0.009	1.00	1.00	1.00
T2	657/645	0.009–0.045	1.14 (0.89, 1.46)	1.00 (0.71, 1.41)	1.01 (0.71, 1.43)
T3	697/644	>0.045	1.34 (1.08, 1.66)	1.15 (0.83, 1.59)	1.10 (0.74, 1.63)

NASH, non-alcoholic steatohepatitis; T1, first tercile; T2, second tercile; T3, third tercile; SFAs, saturated fatty acids; MUFAs, monounsaturated fatty acids; PUFAs, polyunsaturated fatty acids; and UFAs, unsaturated fatty acids.

Model 1: unadjusted; model 2: adjusted for sex, age, and BMI; model 3: model 2 additionally adjusted for ethnicity, marital status, education levels, family income-to-poverty ratio, waist circumference, smoking status, regular activities, use of oral corticosteroid, energy intakes, HEI-2015, ALT, ALP, AST, GGT, hypertension, diabetes mellitus, dyslipidemia, cardiovascular disease, and cancer.

^a^The ratio of UFAs to SFAs was calculated as (PUFAs + MUFAs)/SFAs.

^b^Dietary intakes of fatty acids were adjusted for energy intakes using the residuals method.

### Interactions and stratified analyses

**FIGURE 2:**
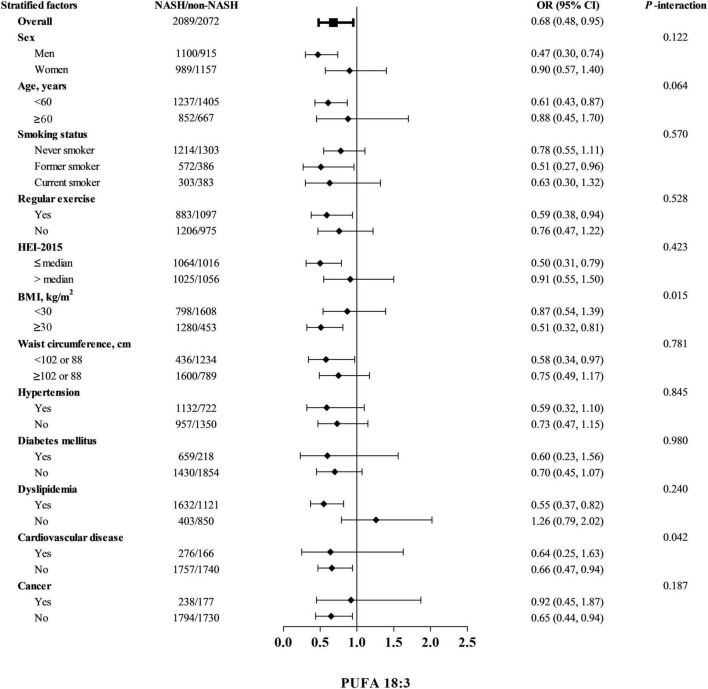
Odds ratios (ORs) and 95% confidence intervals (CIs) of non-alcoholic steatohepatitis by terciles of dietary PUFA 18:3 among controls stratified by covariates. Abbreviations: PUFA, polyunsaturated fatty acid NASH, non-alcoholic steatohepatitis; HEI-2015, healthy eating index-2015; and BMI, body mass index. Cut-off points of waist circumference were 102 cm for men and 88 cm for women. Odds ratios were adjusted for age, sex, BMI, ethnicity, marital status, education levels, family income-to-poverty ratio, waist circumference, smoking status, regular activities, use of oral corticosteroid, energy intakes, HEI-2015, ALT, ALP, AST, GGT, hypertension, diabetes mellitus, dyslipidemia, cardiovascular disease, and cancer. Stratified factors were not included in the corresponding models.

Influences of dietary total PUFAs intakes on risk of NASH stratified by selected potential risk factors were shown in Table 4. Multiplicative interactions were statistically significant between terciles of dietary intakes of total PUFAs and BMI (<30 kg/m^2^ or ≥30 kg/m^2^) on associations with risk of NASH (*p*-interaction = 0.004). Stronger associations of dietary total PUFAs intakes with NASH risk were found in obese participants with a BMI ≥ 30 kg/m^2^ (OR, 95% CI: 0.41, 0.22–0.75), while significant associations could not be observed in non-obese participants with a BMI < 30 kg/m^2^ (OR, 95% CI: 1.00, 0.70–1.43). Null significant multiplicative interactions between other potential risk factors and terciles of dietary total PUFAs were identified on risk of NASH. Similar effects on risk of NASH were also observed between BMI and dietary intakes of PUFA 18:3 (*p*-interaction = 0.015, Figure 2). Moreover, inverse associations of dietary intakes of PUFA 18:3 and NASH risk were stronger among participants without presence of cardiovascular disease (OR, 95% CI: 0.66, 0.47–0.94), compared to their counterparts (OR, 95% CI: 0.64, 0.25–1.63, *p*-interaction = 0.042).

**TABLE 4:** Odds ratios (ORs) and 95% confidence intervals (CIs) of non-alcoholic steatohepatitis by terciles of dietary total polyunsaturated fatty acids among controls stratified by covariates.

Terciles of dietary total PUFAs	NASH/non-NASH	OR (95% CI)^b^			*p*-interaction
		T1	T2	T3	
Sex					0.776
Men	1,100/915	1.00	0.67 (0.35, 1.29)	0.56 (0.34, 0.90)	
Women	989/1,157	1.00	0.87 (0.57, 1.33)	0.79 (0.50, 1.23)	
Age, years					0.083
<60	1,237/1,405	1.00	0.60 (0.35, 1.03)	0.68 (0.47, 0.98)	
≥60	852/667	1.00	1.30 (0.75, 2.24)	0.72 (0.43, 1.21)	
Smoking status					0.217
Never smoker	1,214/1,303	1.00	0.77 (0.49, 1.22)	0.66 (0.43, 1.02)	
Former smoker	572/386	1.00	0.67 (0.27, 1.64)	0.51 (0.25, 1.06)	
Current smoker	303/383	1.00	0.59 (0.29, 1.20)	0.81 (0.40, 1.64)	
Regular exercise					0.053
Yes	883/1,097	1.00	0.72 (0.39, 1.33)	0.53 (0.34, 0.83)	
No	1,206/975	1.00	0.79 (0.46, 1.35)	0.89 (0.53, 1.48)	
HEI-2015					0.997
≤median	1,064/1,016	1.00	0.49 (0.29, 0.82)	0.58 (0.34, 0.98)	
>median	1,025/1,056	1.00	1.34 (0.72, 2.51)	0.83 (0.48, 1.44)	
BMI, kg/m^2^					0.004
<30	798/1,608	1.00	1.10 (0.70, 1.73)	1.00 (0.70, 1.43)	
≥30	1,280/453	1.00	0.48 (0.24, 0.96)	0.41 (0.22, 0.75)	
Waist circumference, cm*^a^*					0.847
<102 or 88	436/1,234	1.00	0.84 (0.50, 1.41)	0.67 (0.43, 1.05)	
≥102 or 88	1,600/789	1.00	0.76 (0.42, 1.38)	0.71 (0.44, 1.12)	
Hypertension					0.862
Yes	1,132/722	1.00	0.90 (0.48, 1.67)	0.46 (0.23, 0.94)	
No	957/1,350	1.00	0.71 (0.46, 1.09)	0.86 (0.61, 1.21)	
Diabetes mellitus					0.966
Yes	659/218	1.00	0.69 (0.25, 1.88)	0.44 (0.12, 1.55)	
No	1,430/1,854	1.00	0.78 (0.46, 1.31)	0.71 (0.47, 1.10)	
Dyslipidemia					0.197
Yes	1,632/1,121	1.00	0.76 (0.42, 1.36)	0.62 (0.43, 0.90)	
No	403/850	1.00	0.84 (0.41, 1.75)	0.95 (0.54, 1.66)	
Cardiovascular disease					0.086
Yes	276/166	1.00	1.80 (0.77, 4.17)	0.86 (0.35, 2.10)	
No	1,757/1,740	1.00	0.72 (0.44, 1.16)	0.67 (0.46, 0.97)	
Cancer					0.305
Yes	238/177	1.00	0.83 (0.28, 2.48)	0.39 (0.14, 1.07)	
No	1,794/1,730	1.00	0.77 (0.46, 1.29)	0.71 (0.49, 1.05)	

NASH, non-alcoholic steatohepatitis; PUFAs, polyunsaturated fatty acids; T1, first tercile; T2, second tercile; T3, third tercile; HEI-2015, healthy eating index-2015; and BMI, body mass index.

^a^Cut-off points of waist circumference were 102 cm for men and 88 cm for women.

^b^Adjusted for age, sex, BMI, ethnicity, marital status, education levels, family income-to-poverty ratio, waist circumference, smoking status, regular activities, use of oral corticosteroid, energy intakes, HEI-2015, ALT, ALP, AST, GGT, hypertension, diabetes mellitus, dyslipidemia, cardiovascular disease, and cancer. Stratified factors were not included in the corresponding models.

## Discussion

In this nationally representative study with 4,161 adults in the United States, we explored the associations between risk of NASH and dietary intakes of fatty acids, including total SFAs, MUFAs, PUFAs, their common subtypes, and the ratio of UFAs to SFAs. The dietary intakes of total PUFAs, as well as its subtype of PUFA 18:3, were inversely associated with risk of NASH, independent of several potential covariates, especially in those with obesity (BMI ≥ 30 kg/m^2^). No significant associations were observed between NASH risk and dietary total SFAs, MUFAs, their subtypes as well as the ratio of UFAs to SFAs.

A few studies have explored the relationships between fatty acids and risk of liver diseases or liver-related indices; however, the results were still inconsistent and inconclusive. For example, high abundance of hepatic total SFAs and MUFAs was observed in humans with NAFLD and mice with NASH, which implied that higher total SFAs and MUFAs may be associated with hepatic lipotoxicity and inflammation (25). However, intervention study using diets full with MUFAs showed decreased cholesterol, triglycerides, and increased HDL-cholesterol levels in participants with NAFLD (26). In this study, no significant associations were found between dietary total SFAs, MUFAs, and NASH risk. In addition, an animal study found that moderate intakes of fatty acids with the high ratio of UFAs to SFAs could inhibit liver lipogenesis and steatosis (27), although no significant association was found between the ratio of UFAs to SFAs and NASH risk in our study. Further studies need to be conducted to confirm these findings.

In regard to the relationship between total PUFAs and risk of NAFLD, a systematic review and meta-analysis of 13 studies, consisting of 668 patients with NAFLD, found that total PUFAs or fish oil supplementation may affect serum ALT levels and improve liver function (28). Similar associations could also be observed in other studies of dietary or supplementation with n-3 fatty acids (29–32). However, contradictory results could also be found. A case–control study conducted in 971 Chinese Han adults found that total PUFAs intakes were positively associated with the risk of NAFLD (10). Another cross-sectional study of 233 American children found that dietary long-chain n-3 fatty acids were inversely associated with portal and lobular inflammation, although no significant effects could be found on NASH, which was assessed using serum ALT and histological parameters (33).

The reasons including sample sizes, population, methods of dietary assessment, detection methods of NAFLD may account for diversities among the findings of the aforementioned studies. Particularly, none of those studies have explored the associations of their subtypes with risk of NASH, which was necessary since different fatty acids subtypes exerted different or even opposite effects on liver health (15), due to discrepancies of the length of carbon chain, straight or branched chain, position and numbers of double bonds (34). To the best of our knowledge, this is the first study to investigate associations between dietary total SFAs, MUFAs, PUFAs, their subtypes, the ratio of UFAs to SFAs, and risk of NASH. No significant associations were observed between NASH risk and dietary total SFAs, MUFAs, their subtypes as well as the ratio of UFAs to SFAs. However, dietary intakes of total PUFAs, as well as its subtype of PUFA 18:3, were inversely associated with risk of NASH, independent of several potential covariates, especially in those with obesity (BMI ≥ 30 kg/m^2^). Several biologic mechanisms could explain the favorable associations between dietary intakes of total PUFAs, its subtype of PUFA 18:3, and the risk of NASH. NASH was characterized as lipid deposition and hepatic inflammation. Oxidative stress, insulin resistance, lipid peroxidation, abnormal secretion of proinflammatory cytokines and adipokines, and intestinal dysbiosis played important roles in the occurrence and development of NAFLD (8, 35–38). In line with our findings on the protective impact of total PUFAs on NASH, total PUFAs have been shown to exert anti-inflammatory effects in both *in vitro* and *in vivo* studies. Supplementation with n-3 PUFAs inhibited lipogenesis, attenuated hepatic oxidative stress, decreased inflammation and increased insulin sensitivity, further to preserve hepatic architecture and prevent hepatic steatosis (39). The n-3 PUFAs could downregulate sterol regulatory element-binding protein-1c (SREBP-1c) and upregulate peroxisome proliferator-activated receptor-alpha (PPAR-α) function, and therefore decreased *de novo* lipogenesis and increased free fatty acids oxidation, improved the biochemical and ultrasonographic manifestations of patients with NAFLD (40). Rats fed with a high-fat, high-calorie solid diet were observed with an increased expression of hepatic adiponectin and PPAR-α, a reduction of tumor necrosis factor-alpha (TNF-α), and therefore an improvement of fatty liver and the degree of liver injury after supplementation of n-3 PUFAs (41).

Polyunsaturated fatty acid 18:3, so called α-linolenic acid, was a kind of important n-3 PUFAs with anti-inflammatory and antioxidant effects for the human body. In a 6-month, randomized, placebo-controlled, double-blind trial, supplementation of n-3 PUFAs impacted on plasma lipid profile in patients with NASH, which was specific in reduction of triglycerides, and therefore improved plasma lipidomic and hepatic proteomic markers of lipogenesis, mitochondrial functions and endoplasmic reticulum stress (42, 43). Further mechanisms about effects of PUFA 18:3 on inflammation need to be certified in future studies.

There may be several kinds of common risk factors and links between overweight, obesity, other related metabolic diseases and NAFLD (44). In our study, an inverse association of dietary intakes of PUFA 18:3 with NASH was observed only among participants without cardiovascular disease. Increasing dietary intakes of PUFA 18:3 may reduce the risk of NASH in participants without presence of cardiovascular disease. The BMI was another risk factor for NASH, and decreases in hepatic fat content were partially attributed to favorable changes in BMI (45). Interactions could be found between dietary PUFAs and obesity on NASH risk in our study. Stronger associations of dietary PUFAs intakes with NASH risk were found in participants with obesity rather than those without obesity, suggesting that obese people might benefit more from increasing dietary intakes of PUFAs.

Our study has notable strengths. First, our observational study was based on a national, representative population with large sample size in the United States. Complex sampling design as well as appropriate sample weight method we conducted increased the reliability and generalizability of our findings. In addition, NASH was diagnosed by transient elastography, which was a non-invasive and fast method with high sensitivity and specificity by comparison with liver biopsy (17–19).

Several limitations need to be considered. First, although we adjusted for multiple potential confounders, including demographic information, lifestyle, medication use and history of chronic diseases, residual confounding cannot be eliminated fully. Furthermore, stratified analysis performed in our study may result in potential statistical power loss, it was desirable to conduct studies with larger sample sizes to validate our finding in the future. In addition, since the progression of NASH was long, dietary assessment in a long term was more appropriate to explore the relationships between dietary intakes of fatty acids and risks of NASH. However, in the analysis of this study, dietary intakes of fatty acids were assessed with a 24-h food recall method in two inconsecutive days, future studies with repeated dietary assessment in a long term are warranted. Moreover, restricted by the observational study design, we cannot definitively exclude the possibility that our findings may be affected by residual confounding and reverse causality. Further prospective or interventional studies need to be carried out to verify our finding about associations between dietary fatty acids and NASH.

## Conclusion

In conclusion, inverse associations were observed between dietary intakes of total PUFAs, as well as its subtype of PUFA 18:3, and risk of NASH after adjusting potential confounders. Further large prospective studies need to be conducted to confirm our findings.

## Data availability statement

The original contributions presented in this study are included in the article/Supplementary material, further inquiries can be directed to the corresponding authors.

## Ethics statement

The studies involving human participants were reviewed and approved by National Center for Health Statistics. The patients/participants provided their written informed consent to participate in this study.

## Author contributions

Z-YL and H-LZ created the analytic design. Z-YL performed data extraction. X-TL, Z-YL, Y-DW, and T-TZ analyzed the data. X-TL drafted the manuscript. X-TL, Z-YL, and H-LZ critically revised the manuscript. All authors read and approved the final manuscript.

## Conflict of interest

The authors declare that the research was conducted in the absence of any commercial or financial relationships that could be construed as a potential conflict of interest.

## Publisher’s note

All claims expressed in this article are solely those of the authors and do not necessarily represent those of their affiliated organizations, or those of the publisher, the editors and the reviewers. Any product that may be evaluated in this article, or claim that may be made by its manufacturer, is not guaranteed or endorsed by the publisher.

## Footnotes

1
https://www.cdc.gov/nchs/nhanes/

## Abbreviations

NAFLD: non-alcoholic fatty liver disease
NASH: non-alcoholic steatohepatitis
SFA: saturated fatty acid
UFA: unsaturated fatty acid
MUFA: monounsaturated fatty acid
PUFA: polyunsaturated fatty acid
NHANES: national health and nutrition examination survey
MEC: mobile examination center
CAP: controlled attenuation parameter
HEI-2015: healthy eating index-2015
BMI: body mass index
ALT: alanine aminotransferase
ALP: alkaline phosphatase
AST: aspartate aminotransferase
GGT: gamma-glutamyl transferase
SE: standard error
OR: odds ratio
CI: confidence interval
SREBP-1c: sterol regulatory element-binding protein-1c
PPAR- α: peroxisome proliferator-activated receptor-alpha
TNF- α: tumor necrosis factor-alpha
NLRP3: NOD-like receptor protein 3.
