# Supplementary material for: Dietary fatty acids and risk of non-alcoholic steatohepatitis: A national study in the United States
Source: Front Nutr. 2022 Jul 26;9:952451. doi: 10.3389/fnut.2022.952451 (PMC9360798; doi:10.3389/fnut.2022.952451)
Supplement: Supplementary file 1 [file Data_Sheet_1.PDF]

Supplementary Material

Supplementary Table 1. Basic characteristics by the included and the excluded participants[a].

	Included participants (*n* = 4161)	Excluded participants (*n* = 1695)	*P*
Age, years	47.5 ± 0.8	46.8 ± 0.7	0.578
Sex, *n* (%)			0.512
Men	2015 (48.6)	825 (46.8)	
Women	2146 (51.4)	870 (53.2)	
Ethnicity, *n* (%)			0.535
Non-Hispanic white	1442 (62.6)	590 (60.2)	
Non-Hispanic black	971 (11.4)	372 (11.4)	
Mexican American	582 (9.1)	210 (9.1)	
Others	1166 (17.0)	523 (19.3)	
Marital status, *n* (%)			0.091
Married/living with partner	2340 (60.0)	912 (60.8)	
Separated/divorced/widowed	885 (18.0)	420 (18.2)	
Never married	711 (18.1)	295 (19.0)	
Education levels, *n* (%)			0.030
Less than high school	753 (10.4)	364 (12.6)	
High school or equivalent	956 (27.0)	369 (23.9)	
College or above	2225 (58.7)	889 (61.2)	
Family income-to-poverty ratio, *n* (%)			0.279
<1.3	1039 (20.0)	382 (20.8)	
1.3–3.5	1552 (37.6)	571 (33.5)	
>3.5	1068 (42.4)	409 (45.7)	
BMI, kg/m^2	29.8 ± 0.3	29.3 ± 0.3	0.098
Waist circumference, cm	100.7 ± 0.8	99.3 ± 0.9	0.121
Smoking status, *n* (%)			0.116
Never smoker	2517 (60.2)	980 (54.7)	
Former smoker	958 (24.1)	380 (24.1)	
Current smoker	686 (15.7)	335 (21.2)	
Regular exercise, *n* (%)	1980 (53.8)	756 (54.8)	0.723
**Laboratory parameters, IU/L**			
ALT	22.5 ± 0.3	24.1 ± 1.1	0.165
ALP	77.4 ± 0.7	76.5 ± 1.3	0.497
AST	21.5 ± 0.2	24.3 ± 0.7	0.001
GGT	27.8 ± 0.6	35.3 ± 1.3	<0.001

**Prevalence of chronic diseases, *n* (%)**			
Hypertension	1854 (38.3)	730 (40.8)	0.310
Diabetes mellitus	877 (15.4)	302 (13.7)	0.106
Dyslipidemia	2753 (65.0)	872 (57.4)	<0.001
Cardiovascular disease	442 (8.3)	249 (12.0)	0.024
Cancer	415 (10.6)	173 (10.5)	0.110

Abbreviations: BMI, body mass index; ALT, alanine aminotransferase; ALP, alkaline phosphatase; AST, aspartate aminotransferase; GGT, gamma-glutamyl transferase.

Data were presented as weighted mean ± SE or counts (weighted frequencies).

[a]: Only for adults (≥18 years).

Supplementary Table 2. Components and scoring standards for Healthy Eating Index-2015.

Components	Maximum score	Standard for maximum score	Standard for 0 score
Total fruits	5	≥0.8 cup equivalents/1000 kcal	No fruit
Whole fruits	5	≥0.4 cup equivalents/1000 kcal	No whole fruit
Total vegetables	5	≥1.1 cup equivalents/1000 kcal	No vegetables
Greens and beans	5	≥0.2 cup equivalents/1000 kcal	No dark green vegetables or legumes
Whole gains	10	≥1.5 oz equivalents/1000 kcal	No whole gains
Dairy	10	≥1.3 cup equivalents/1000 kcal	No dairy
Total protein foods	5	≥2.5 oz equivalents/1000 kcal	No protein foods
Seafood and plant proteins	5	≥0.8 cup equivalents/1000 kcal	No seafood or plant proteins
Fatty acids	10	(PUFAs + MUFAs)/SFAs ≥2.5	(PUFAs + MUFAs)/SFAs ≤1.2
Refined grains	10	≤1.8 oz equivalents/1000 kcal	≥4.3 oz equivalents/1000 kcal
Sodium	10	≤1.1 gram/1000 kcal	≥2.0 gram/1000 kcal
Added sugars	10	≤6.5% of energy	≥26% of energy
Saturated fats	10	≤8% of energy	≥16% of energy

Abbreviations: PUFAs, polyunsaturated fatty acids; MUFAs, monounsaturated fatty acids; SFAs, saturated fatty acids.

Supplementary Table 3. Odds ratios (ORs) and 95% confidence intervals (CIs) of nonalcoholic steatohepatitis by terciles of dietary intakes of fatty acids among non-nonalcoholic steatohepatitis in participants who did not use oral corticosteroid (n = 4118).

Dietary intakes	NASH/non-NASH	Levels, gram/day[b]	Model 1	Model 2	Model 3
**Total SFAs**					
T1	622/682	≤19.78	1.00	1.00	1.00
T2	739/687	19.78–25.56	1.09 (0.84, 1.40)	1.04 (0.78, 1.39)	0.97 (0.68, 1.37)
T3	707/680	>25.56	1.11 (0.85, 1.44)	0.86 (0.60, 1.23)	0.80 (0.54, 1.19)
**Total MUFAs**					
T1	710/684	≤22.09	1.00	1.00	1.00
T2	660/682	22.09–27.23	0.79 (0.60, 1.05)	0.69 (0.51, 0.94)	0.69 (0.49, 0.96)
T3	698/683	>27.23	0.91 (0.71, 1.15)	0.63 (0.43, 0.92)	0.67 (0.44, 1.02)
**Total PUFAs**					
T1	718/680	≤14.34	1.00	1.00	1.00
T2	698/683	14.34–19.24	0.91 (0.67, 1.23)	0.73 (0.50, 1.06)	0.76 (0.47, 1.24)
T3	652/686	>19.24	0.75 (0.56, 1.01)	0.61 (0.46, 0.80)	0.67 (0.46, 0.97)
**The ratio of UFAs to SFAs[a]**					
≤1.2	226/224	-	1.00	1.00	1.00
1.2–2.5	1547/1488	-	0.96 (0.74, 1.25)	0.91 (0.68, 1.21)	1.04 (0.68, 1.60)
≥2.5	295/337	-	0.93 (0.64, 1.36)	0.88 (0.69, 1.11)	1.07 (0.68, 1.68)
**Subtypes of fatty acids**					
**SFA 4:0**					
T1	634/679	≤0.234	1.00	1.00	1.00
T2	767/680	0.234–0.462	1.17 (0.92, 1.48)	1.28 (0.97, 1.69)	1.28 (0.97, 1.69)
T3	657/676	>0.462	0.99 (0.73, 1.34)	1.08 (0.75, 1.55)	1.11 (0.72, 1.71)
**SFA 6:0**					
T1	656/677	≤0.161	1.00	1.00	1.00
T2	739/673	0.161–0.307	1.07 (0.88, 1.31)	1.12 (0.85, 1.49)	1.08 (0.81, 1.43)
T3	659/674	>0.307	0.97 (0.69, 1.37)	0.98 (0.67, 1.42)	1.01 (0.67, 1.54)
**SFA 8:0**					
T1	681/682	≤0.140	1.00	1.00	1.00
T2	697/682	0.140–0.247	0.92 (0.75, 1.14)	0.99 (0.76, 1.28)	1.02 (0.75, 1.39)
T3	685/679	>0.247	1.00 (0.75, 1.34)	1.08 (0.79, 1.47)	1.15 (0.81, 1.63)
**SFA 10:0**					
T1	659/683	≤0.290	1.00	1.00	1.00
T2	754/682	0.290–0.518	1.05 (0.88, 1.26)	1.12 (0.87, 1.45)	1.14 (0.85, 1.53)
T3	654/682	>0.518	0.92 (0.68, 1.22)	1.02 (0.73, 1.43)	1.08 (0.72, 1.63)
**SFA 12:0**					
T1	690/681	≤0.404	1.00	1.00	1.00

T2	681/686	0.404–0.783	0.93 (0.71, 1.23)	0.97 (0.70, 1.35)	0.92 (0.60, 1.41)
T3	697/679	>0.783	0.97 (0.70, 1.34)	1.02 (0.77, 1.35)	1.02 (0.78, 1.32)
**SFA 14:0**					
T1	647/682	≤1.344	1.00	1.00	1.00
T2	728/687	1.344–2.163	1.01 (0.78, 1.30)	1.02 (0.77, 1.35)	0.98 (0.75, 1.27)
T3	693/680	>2.163	1.00 (0.73, 1.36)	0.92 (0.66, 1.27)	0.89 (0.62, 1.28)
**SFA 16:0**					
T1	630/683	≤11.242	1.00	1.00	1.00
T2	714/685	11.242–14.124	1.11 (0.78, 1.56)	0.96 (0.66, 1.39)	0.98 (0.63, 1.52)
T3	724/681	>14.124	1.12 (0.84, 1.48)	0.78 (0.55, 1.10)	0.73 (0.50, 1.06)
**SFA 18:0**					
T1	613/683	≤4.593	1.00	1.00	1.00
T2	700/684	4.593–6.065	1.03 (0.76, 1.40)	0.91 (0.67, 1.25)	0.90 (0.61, 1.33)
T3	755/682	>6.065	1.20 (0.92, 1.57)	0.84 (0.58, 1.21)	0.75 (0.48, 1.18)
**MUFA 16:1**					
T1	658/684	≤0.775	1.00	1.00	1.00
T2	689/684	0.775–1.132	1.10 (0.88, 1.38)	0.91 (0.69, 1.20)	0.97 (0.68, 1.39)
T3	721/681	>1.132	1.25 (0.98, 1.58)	0.83 (0.60, 1.17)	0.85 (0.56, 1.28)
**MUFA 18:1**					
T1	709/685	≤20.691	1.00	1.00	1.00
T2	669/680	20.691–25.586	0.81 (0.61, 1.07)	0.68 (0.49, 0.94)	0.66 (0.46, 0.95)
T3	690/684	>25.586	0.94 (0.75, 1.20)	0.65 (0.46, 0.92)	0.71 (0.49, 1.02)
**MUFA 20:1**					
T1	694/684	≤0.205	1.00	1.00	1.00
T2	673/682	0.205–0.296	1.01 (0.77, 1.32)	0.83 (0.62, 1.10)	0.85 (0.63, 1.15)
T3	700/683	>0.296	1.03 (0.78, 1.35)	0.72 (0.52, 1.00)	0.79 (0.53, 1.16)
**MUFA 22:1**					
T1	655/643	≤0.008	1.00	1.00	1.00
T2	625/642	0.008–0.023	1.05 (0.88, 1.25)	0.92 (0.78, 1.10)	1.08 (0.84, 1.39)
T3	682/647	>0.023	1.12 (0.95, 1.32)	0.91 (0.66, 1.27)	1.06 (0.73, 1.54)
**PUFA 18:2**					
T1	723/680	≤12.745	1.00	1.00	1.00
T2	686/684	12.745–17.031	0.89 (0.64, 1.24)	0.71 (0.46, 1.09)	0.74 (0.44, 1.23)
T3	659/685	>17.031	0.76 (0.56, 1.04)	0.63 (0.46, 0.86)	0.68 (0.46, 1.01)
**PUFA 18:3**					
T1	686/683	≤1.244	1.00	1.00	1.00
T2	703/682	1.244–1.776	1.09 (0.80, 1.50)	1.01 (0.71, 1.44)	1.10 (0.74, 1.63)
T3	679/684	>1.776	0.83 (0.62, 1.12)	0.65 (0.48, 0.86)	0.67 (0.47, 0.96)
**PUFA 18:4**					
T1	496/478	≤0.001	1.00	1.00	1.00
T2	514/471	0.001–0.004	0.99 (0.67, 1.45)	0.92 (0.62, 1.36)	0.91 (0.57, 1.45)
T3	464/474	>0.004	1.11 (0.75, 1.65)	1.03 (0.65, 1.64)	1.02 (0.61, 1.70)

**PUFA 20:4**					
T1	650/679	≤0.093	1.00	1.00	1.00
T2	691/684	0.093–0.164	1.00 (0.70, 1.43)	0.83 (0.54, 1.27)	0.77 (0.48, 1.26)
T3	726/679	>0.164	1.19 (0.93, 1.53)	0.84 (0.62, 1.15)	0.75 (0.52, 1.07)
**PUFA 20:5**					
T1	644/654	≤0.006	1.00	1.00	1.00
T2	715/659	0.006–0.013	1.18 (0.93, 1.49)	1.03 (0.85, 1.25)	1.01 (0.82, 1.25)
T3	636/658	>0.013	1.12 (0.83, 1.50)	0.93 (0.65, 1.35)	0.99 (0.68, 1.43)
**PUFA 22:5**					
T1	673/665	≤0.014	1.00	1.00	1.00
T2	673/668	0.014–0.023	1.03 (0.73, 1.47)	0.93 (0.63, 1.36)	0.85 (0.58, 1.23)
T3	688/665	>0.023	1.09 (0.81, 1.46)	0.82 (0.56, 1.20)	0.82 (0.55, 1.23)
**PUFA 22:6**					
T1	597/638	≤0.009	1.00	1.00	1.00
T2	652/639	0.009–0.045	1.15 (0.89, 1.48)	1.00 (0.71, 1.42)	1.01 (0.71, 1.43)
T3	689/636	>0.045	1.34 (1.07, 1.67)	1.14 (0.82, 1.60)	1.11 (0.75, 1.65)

Abbreviations: NASH, nonalcoholic steatohepatitis; T1, first tercile; T2, second tercile; T3, third tercile; UFAs, unsaturated fatty acids; SFAs, saturated fatty acids; MUFAs, monounsaturated fatty acids; PUFAs, polyunsaturated fatty acids.

Model 1: unadjusted; model 2: adjusted for sex, age, and BMI; model 3: model 2 additionally adjusted for ethnicity, marital status, education levels, family income-to-poverty ratio, waist circumference, smoking status, regular activities, use of oral corticosteroid, energy intakes, HEI-2015, ALT, ALP, AST, GGT, hypertension, diabetes mellitus, dyslipidemia, cardiovascular disease and cancer.

a: The ratio of UFAs to SFAs was calculated as (PUFAs + MUFAs)/SFAs.

b: Dietary intakes of fatty acids were adjusted for energy intakes using the residuals method.
